# Quality of life and psychosocial adjustment: the moderator role of anxiety symptoms in persons with temporal lobe epilepsy due to hippocampal sclerosis

**DOI:** 10.1192/j.eurpsy.2023.737

**Published:** 2023-07-19

**Authors:** E. M. Lima, J. Gois, S. Vincentiis, K. D. R. Valente

**Affiliations:** 1 Department of Psychiatry, University of Sao Paulo, Sao Paulo, SP, Brazil

## Abstract

**Introduction:**

Persons with temporal lobe epilepsy due to hippocampal sclerosis (TLE-HS) have a high frequency of psychiatric disorders, namely depression and anxiety. In addition, poor quality of life (QOL) and impairments in psychosocial adjustment are frequently reported. Despite the increasing number of studies aiming to identify predictors of poorer QOL, the role of depressive and anxiety symptoms remains poorly understood.

**Objectives:**

This study aimed to evaluate: (**1**) if psychosocial adjustment predicts worse QOL; (**2**) the relationship between psychosocial adjustment and QOL by concurrently examining the role of depressive and anxiety symptoms.

**Methods: Participants:**

Thirty-five persons with TLE-HS ranging from 18 to 60 years old (mean age 39.82 SD 9.05; 20 men 57.14%) followed in a tertiary outpatient center underwent neurologic and psychiatric assessments.

**Assessments:**

The psychiatry interview was performed using the Structured Clinical Interview for Diagnostic and Statistical Manual of Mental Disorders (SCID). Psychosocial adjustment was assessed with the total score of the Social Adjustment Scale (SAS) (SAS Overall). The QOL was evaluated by the total score of the Quality of Life in Epilepsy-31 Inventory (Overall QOLIE-31), the most frequently used instrument in assessing QOL in epilepsy. The Beck Depression Inventory (BDI) and the State-Trait Anxiety Inventory (STAI-X) were used to assess depressive and anxiety symptoms, respectively.

**Analyses:**

Prediction analysis was performed to evaluate the impact of psychosocial adjustment on QOL (simple linear regression). Simple moderation models were used to examine the moderation effect of depressive and anxiety symptoms on the association between PA and QOL (Figure 1). We used SPSS (version 29 IBM Corp) and PROCESS Macro (version 4.1. for SPSS) to perform regression and moderation analyses (Figures 2 and 3), respectively.

**Results:**

Poor psychosocial adjustment (higher scores on SAS) impacted on poor QOL (lower scores on QOLIE-31) (R=0.39; R2=0.15; adjusted R2= 0.12; B=-0.39; t=-2.28; p=0.03). The severity of anxiety symptoms (Trait and State; coefficient=-0.64; t=-2.01; p=0.05 and coefficient=-1.17; t=-2.20; p=0.03, respectively), but not the severity of depressive symptoms (coefficient=0.77; t= 1.37; p=0.18), moderated the relationship between psychosocial adjustment and QOL.

**Image:**

**Figure:**
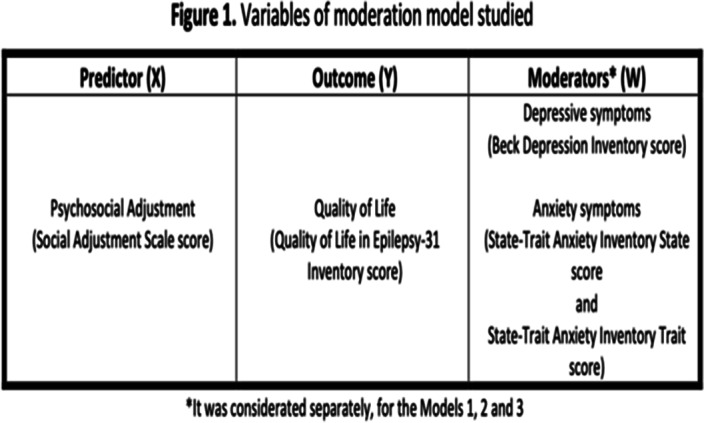

**Image 2:**

**Figure:**
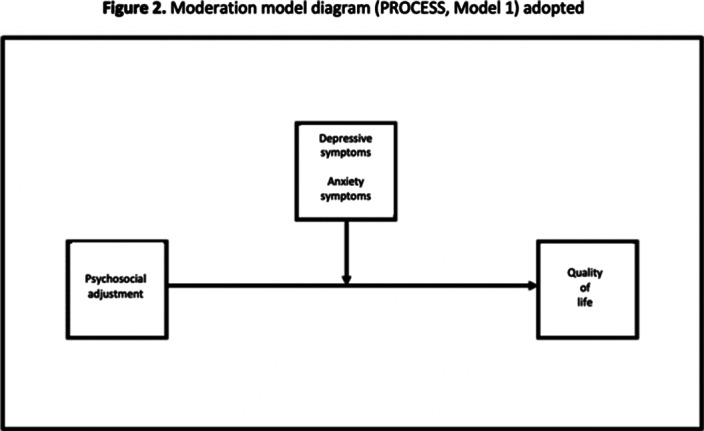

**Image 3:**

**Figure:**
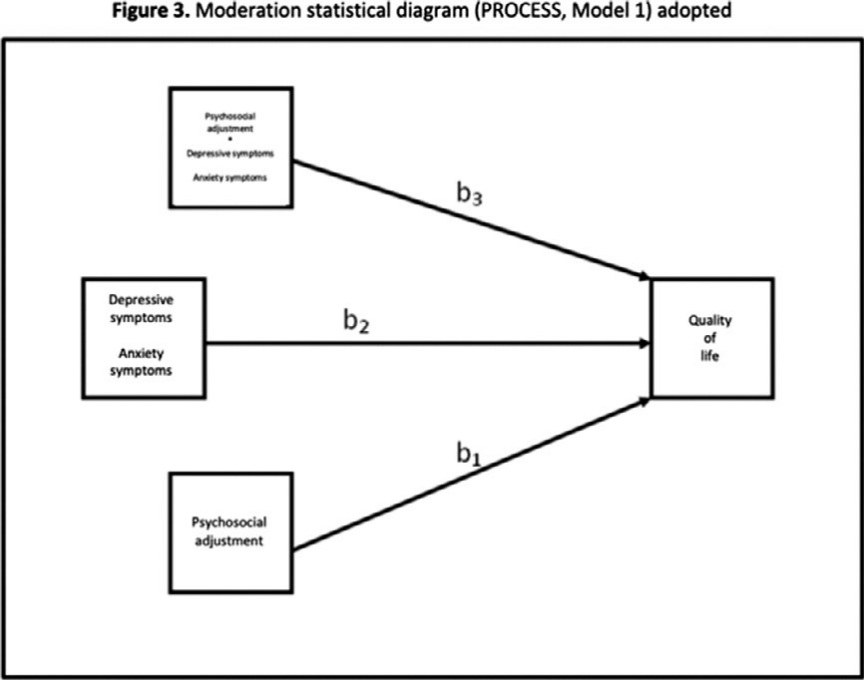

**Conclusions:**

Psychosocial adjustment is a predictor of QOL in TLE-HS. Anxiety symptoms moderate this relationship between psychosocial adjustment and QOL. Consequently, higher anxiety symptoms are associated with worse psychosocial adjustment and quality of life.

**Disclosure of Interest:**

None Declared

